# Emergency department visits and hospitalizations by tube-fed nursing home residents with varying degrees of cognitive impairment: a national study

**DOI:** 10.1186/1471-2318-14-35

**Published:** 2014-03-20

**Authors:** Caroline E Stephens, Nathan Sackett, Prasanthi Govindarajan, Sei J Lee

**Affiliations:** 1 Department of Community Health Systems, University of California San Francisco, 2 Koret Way, #N531E, San Francisco, CA 94143-0608, USA; 2 Division of Geriatrics, Palliative & Extended Care, UCSF/San Francisco VA Medical Center, 4150 Clement St, San Francisco, CA 94121, USA; 3 UC Berkeley/UCSF Joint Medical Program, 570 University Hall #1190, Berkeley, CA 94720, USA; 4 Department of Emergency Medicine, University of California San Francisco, 505 Parnassus Ave, San Francisco, CA 94143, USA

**Keywords:** Tube feeding, Cognitive impairment, Nursing home, Emergency department, Hospitalization, Ambulatory care sensitive

## Abstract

**Background:**

Numerous studies indicate that the use of feeding tubes (FT) in persons with advanced cognitive impairment (CI) does not improve clinical outcomes or survival, and results in higher rates of hospitalization and emergency department (ED) visits. It is not clear, however, whether such risk varies by resident level of CI and whether these ED visits and hospitalizations are potentially preventable. The objective of this study was to determine the rates of ED visits, hospitalizations and potentially preventable ambulatory care sensitive (ACS) ED visits and ACS hospitalizations for long-stay NH residents with FTs at differing levels of CI.

**Methods:**

We linked Centers for Medicare and Medicaid Services inpatient & outpatient administrative claims and beneficiary eligibility data with Minimum Data Set (MDS) resident assessment data for nursing home residents with feeding tubes in a 5% random sample of Medicare beneficiaries residing in US nursing facilities in 2006 (n = 3479). Severity of CI was measured using the Cognitive Performance Scale (CPS) and categorized into 4 groups: None/Mild (CPS = 0-1, MMSE = 22-25), Moderate (CPS = 2-3, MMSE = 15-19), Severe (CPS = 4-5, MMSE = 5-7) and Very Severe (CPS = 6, MMSE = 0-4). ED visits, hospitalizations, ACS ED visits and ACS hospitalizations were ascertained from inpatient and outpatient administrative claims. We estimated the risk ratio of each outcome by CI level using over-dispersed Poisson models accounting for potential confounding factors.

**Results:**

Twenty-nine percent of our cohort was considered “comatose” and “without any discernible consciousness”, suggesting that over 20,000 NH residents in the US with feeding tubes are non-interactive. Approximately 25% of NH residents with FTs required an ED visit or hospitalization, with 44% of hospitalizations and 24% of ED visits being potentially preventable or for an ACS condition. Severity of CI had a significant effect on rates of ACS ED visits, but little effect on ACS hospitalizations.

**Conclusions:**

ED visits and hospitalizations are common in cognitively impaired tube-fed nursing home residents and a substantial proportion of ED visits and hospitalizations are potentially preventable due to ACS conditions.

## Background

Cognitively impaired nursing home residents are a large and growing segment of the Medicare population who often require acute care services such as emergency department (ED) visits and hospitalizations. In 2009, 68% of all nursing home residents had some degree of cognitive impairment (CI) [1], with the number of persons with CI expected to triple by 2050 [2]. Further, cognitively impaired nursing home residents are at increased risk for potentially preventable hospitalizations and ED visits [3-5]. Thus, the population of cognitively impaired nursing home residents will continue to grow rapidly and will likely continue to require frequent acute care services.

Unfortunately, CI is usually progressive, frequently leading to feeding problems and dysphagia [6]. When this occurs, providers and families face the difficult decision regarding feeding tube (FT) placement for artificial hydration and nutrition. Although there is no evidence that the use of FTs improves clinical outcomes or survival in this patient group [7-11], the practice is incredibly common with as many as 34% of nursing home (NH) residents with severe CI having a FT [12]. This may be due in part to the perception by physicians and families that FT placement has minimal risks or burdens [13,14]. However, studies suggest that NH residents with FTs are at increased risk for hospitalizations [15] and ED visits [3,4], compared to those without FTs. One recent study found that 26% of tube fed NH residents had a potentially preventable ED visit [4]. Frequent hospital transfers in this already vulnerable population often leads to greater cognitive and functional decline, delirium, iatrogenic complications and death [16,17].

Studies to date on the relationship between ED visits and hospitalizations in NH residents with FTs have focused on those with advanced dementia. Little is known about whether the risk of acute care utilization in NH residents with FTs varies by the severity of their CI and whether these ED visits and hospitalizations are potentially preventable. Such information may give families and providers a clearer picture of the likely burdens associated with FT use based on cognitive status and allow them to make a more informed care decision about initial feeding tube placement. Thus, our objective was to determine the rates of ED visits and hospitalizations by differing levels of CI among a national random sample of NH residents with FTs. Further, we examined the rates of potentially preventable ED visits and hospitalizations by determining the rates of Ambulatory Care Sensitive (ACS) ED visits and ACS hospitalizations.

## Methods

### Study design

We conducted a retrospective cohort study utilizing 2006 Centers for Medicare and Medicaid Services (CMS) inpatient and outpatient administrative claims and beneficiary eligibility data linked with nursing home resident assessment data. This research study was approved by the University of California, San Francisco’s Committee on Human Research.

### Study setting and population

We examined a 5% random sample of all Medicare fee-for-service beneficiaries over the age of 65 residing in U.S. nursing homes in 2006 from the Centers for Medicare and Medicaid Services (CMS) Chronic Condition Data Warehouse standard analytic file (for CMS standard sampling strategy: https://www.ccwdata.org/web/guest/user-documentation). NH residence was determined by the presence of a Minimum Data Set (MDS) 2.0 assessment in 2006. We focused on NH residents with FTs, only including residents whose MDS item K5b, “Nutritional Approaches: Feeding tube?” was checked. We excluded residents receiving hospice (n = 77) and those with missing cognitive or covariate data (n = 53), leading to our final analytic sample of 3479 NH residents with FTs. Since we were specifically interested in the rates of acute care utilization by long stay NH residents, we measured outcomes after a 90-day blackout window after initial NH assessment to exclude short-stay residents.

### Primary predictor

The primary predictor of interest was level of CI as defined by the Cognitive Performance Scale (CPS). The CPS is a 7-point hierarchical scale derived from the Minimum Data Set (MDS) that rates CI from 0 (no impairment) to 6 (very severe impairment) and has been widely used as a valid measure of cognition in previous studies of NH residents [18-20]. The 5 MDS items used in this scale include: Comatose Status, Short-Term Memory, Cognitive Skills for Daily Living, Making Self Understood by Others, and Activities of Daily Living (ADL) Self-Performance in Eating. Previous validation studies suggest that the CPS is tightly correlated with other cognitive scales, including the Mini-Mental Status Exam (MMSE) [18,19,21-23].

For this study, we categorized severity of CI into 4 groups: No/Mild (0, 1); Moderate (2, 3); Severe (4, 5); Very Severe (6). NH residents in the No/Mild category (CPS 0,1) are considered intact or borderline intact with Folstein Mini-Mental Status Exam (MMSE) scores of 22 to 25 [24]. NH residents in the Moderate impairment category (CPS 2,3) have been shown to have MMSE scores of 15 to 19. NH residents in the Severe impairment category (CPS 4,5) have been shown to have MMSE scores of 5 to 7. NH residents in the Very Severe category (CPS 6) have profound impairment and are characterized as being “comatose” or showing “no discernible consciousness” with an MMSE of 0 to 4. Since our focus was on the majority of tube-fed NH residents with CI, we chose the Moderate CI group as our reference category rather than the No/Mild CI group.

### Primary outcomes

Inpatient and outpatient claims data were used to examine the rates of the following 4 outcomes: *1) ED Visit; 2) ACS ED Visit; 3) Hospitalization; 4) ACS Hospitalization.* The *ED visit* outcome was defined as any ED visit that did not result in a hospitalization using the Outpatient Standard Analytic File (SAF) (revenue center codes 0450–0459 and 0981). The *ACS ED Visit* outcome was defined as any ED visit that did not result in a hospitalization, but had an ACS condition listed as the primary (final) reason for the ED stay. To classify visits as ACS, we used the Agency for Healthcare Research and Quality’s (AHRQ) “Prevention Quality Indicators” (PQIs) that aim to identify ACS conditions based on ICD-9 codes [25]. This AHRQ methodology is widely accepted and has been used extensively in previous literature as an indicator of potentially preventable hospitalizations and ED visits [26-28]. These are standardized measures based on ACS condition classifications [29,30] and have been validated and commonly used in prior studies of ACS acute care utilization among elderly long term care residents [28,31-33]. The Hospitalization outcome was defined as any hospitalization as identified in the Medicare Provider Analysis and Review (MEDPAR) file (short, long, skilled nursing facility stay indicator code = S). The *ACS Hospitalization* outcome was defined as any hospitalization for an ACS condition listed as the primary reason for the hospital stay in the MEDPAR file. If K5b (feeding tube present) was ‘yes’ on any qualifying MDS assessment then the resident was considered to be at risk for any of the above outcomes through death or the end of calendar year 2006.

### Possible confounding factors

We examined several widely used MDS resident variables to adjust for potential differences in sociodemographic characteristics and disease severity [20,34-39]. Selected characteristics included sociodemographic variables (age, sex, race/ethnicity, marital status), previously diagnosed medical illnesses or conditions (stroke, any stage 2+ pressure ulcer), level of ADL impairment, and certain treatments/preferences (Do Not Resuscitate order, any use of a urinary catheter). Age was coded into 3 categories: 65 to 75 years, 76 to 85 years, and 86+ years. Similar to previous studies, we included race/ethnicity as a categorical variable (as captured in the MDS) as evidence suggests that FT placement differs by race/ethnicity [9]. Due to small numbers of Hispanics, Asians, Pacific Islanders, and Native Americans, these race/ethnicity categories were collapsed into a single category of “other”. Since no prior investigations have specifically examined a cohort of only tube-fed residents (vs general NH population), we selected ADLs and constructed ADL categories that were both clinically meaningful and reasonably distributed across our 5% national random sample of tube-fed nursing home residents. Specifically, level of Activities of Daily Living (ADL) Impairment was determined by summing the observed physical function ratings in each of 5 ADLs (Eating, Toileting, Bathing, Dressing and Transferring) examined in the MDS. Each ADL was rated on a scale of 0 (total independence) to 4 (total dependence), leading to a total ADL score ranging from 0 to 20. We collapsed the ADL data into the following 4 categories: Independent- Limited ADL Assistance (score 0–12); Extensive ADL Assistance (score 13–17); Total Dependence in Most ADLs (score 18–19); Total Dependence in all ADLs (score 20). The MDS-Changes in Health, End-stage disease and Symptoms and Signs (CHESS) scale, was used to account for overall health status [40].

### Data analysis

Descriptive statistics were used to characterize the sample of NH residents by the severity of their CI. Frequency, percent and measures of central tendency were employed to summarize the characteristics of the sample and evaluate the data. Frequency counts, chi-square and Kruskal-Wallis test p-values were calculated to compare the proportion of patients by the severity of their CI. Nominal variables have a chi-square p-value and ordered variables have a nonparametric Kruskal-Wallis test p-value.

We then examined the distribution of possible confounding factors and outcomes across levels of CI. Descriptive statistics suggested overdispersed count data leading us to utilize overdispersed Poisson models to estimate the unadjusted and adjusted incident rate ratio (IRR) of each of the outcomes by level of CI. Since NH residents with MDS evaluations early in the year are at risk for acute care needs for a longer time period than NH residents with MDS evaluations later in the year, we included an exposure offset in our models, and present our outcomes in person-years of observation with 95% confidence intervals. All analyses were performed using SAS version 9.2 (SAS Institute, Inc., Cary, NC).

## Results

In our study, we found 3479 NH residents had feeding tubes, suggesting that nearly 70,000 NH residents in the US were tube-fed in 2006. Table 1 shows the sociodemographic characteristics of NH residents with FTs by level of CI. Twenty-nine percent (1003/3479) of NH residents with FTs had Very Severe CI and were considered “comatose” or having “no discernible consciousness”. Since our analytic sample represents a random 5% sample of Medicare beneficiaries, this result suggests that over 20,000 US NH residents with FTs were “comatose” or had “no discernible consciousness” in 2006. Most participants were female (54%), and almost 20% were African American. NH residents with FTs in this study sample also had profound ADL impairment with 42% reporting dependence in all ADLs, representing nearly 30,000 US NH residents who are completely dependent in all ADLs (toileting, transferring, eating, dressing and bathing).

**Table 1:** **Characteristics of nursing home residents with feeding tubes and characteristics by level of cognitive impairment (CI)**^1^*

**Characteristic**	**Total sample (n = 3479), %**	**No/Mild CI (n = 785), %**	**Mild/Moderate CI (n = 1109), %**	**Moderate/Severe CI (n = 582), %**	**Very severe CI (n = 1003), %**
**Sociodemographics**					
Sex					
Male	46	48	49	46	40
Female	54	52	51	54	60
Race/Ethnicity					
White	73	86	79	70	58
Black	19	10	16	20	28
Other	8	4	5	10	14
Age group					
65-75	27	36	25	24	25
76-85	47	47	49	47	44
86+	26	17	25	30	31
Married	40	44	41	39	36
**Resident diagnoses & conditions**					
CVA	38	18	36	49	51
Stage 2+ pressure ulcer	37	29	35	33	48
ADL impairment level^2^					
Independent-limited ADL	9	28	8	1	0
Assistance (0–12)					
Extensive ADL assistance (13–17)	32	49	44	30	5
Total dependence in most ADLs (18–19)	17	11	21	25	12
Total dependence in all ADLs (20)	42	13	26	43	83
CHESS category					
Mild (0)	20	15	19	22	26
Moderate (1–3)	75	85	75	73	69
Severe (4–5)	4	<1	6	5	5
**Treatments & preferences**					
Do not resuscitate order	33	24	33	37	39
Any urinary catheter use	48	39	45	53	57

^1^Cognitive status measured by the Cognitive Performance Scale (CPS) – No/Mild (0, 1); Mild/Moderate (2, 3); Moderate/Severe (4, 5), Very Severe (6).

^2^ADL Impairment Level is a composite measure of 0–4 level of impairment for each ADL of transferring, dressing, toileting, bathing, and eating yielding a total possible score of 20 (representing total dependence in ADLs). We defined 4 categories of ADL Impairment: Independent- Limited ADL Assistance (score 0–12); Extensive ADL Assistance (score 13–17); Total Dependence in Most ADLs (score 18–19); Total Dependence in all ADLs (score 20).

*All characteristics were significantly different across all 4 levels of CI (p < .0001).

Table 2 shows the results of our overdispersed Poisson regression that modeled the rates of ED visits and ACS ED Visits among this national random sample of NH residents with feeding tubes. Ten percent of NH residents with FTs required an ED visit and 2.3% had a potentially preventable ACS ED visit in 2006. Twenty-four percent (79/335) of all ED visits by NH residents with FTs were considered potentially preventable and due to an ACS condition. Overdispersed poisson models revealed that NH residents with moderate/severe CI had significantly higher rates of ED and ACS ED visits compared to those with mild/moderate CI [IRR = 1.42 (1.16-1.74) and IRR = 1.80 (1.35-2.41), respectively]. In contrast, NH residents with very severe CI had similar adjusted rates of ED visits and ACS ED visits as NH residents with mild/moderate CI [IRR = 1.05 (.85-1.30) and IRR = 1.17 (.87-1.57), respectively].

**Table 2:** Rates of ED Visits and ACS ED visits according to the characteristics of nursing home residents with feeding tubes

**Characteristic**	**ED visit rate, no. (%)**	**ED visit incident rate ratios (95%confidence interval)**		**ACS ED visit rate, no. (%)**	**ACS ED incident rate ratios (95% confidence interval)**	
		**Unadjusted**	**Adjusted**^**+**^		**Unadjusted**	**Adjusted**^**+**^
Total	335 (9.6)			79 (2.3)		
Severity of cognitive impairment^1^						
No/Mild	82 (10.4)	1.00 (.83-1.20)	.92 (.76-1.11)	24 (3.1)	1.79 (1.37-2.33)***	1.84 (1.40-2.41)***
Mild/Moderate	105 (9.5)	Ref	Ref	21 (1.9)	Ref	Ref
Moderate/Severe	65 (11.2)	1.35 (1.10-1.65)**	1.42 (1.16-1.74)**	14 (2.4)	2.02 (1.50-2.71)***	1.80 (1.35-2.41)***
Very severe	83 (8.3)	.89 (.73-1.08)	1.05 (.85-1.30)	20 (2.0)	1.42 (1.07-1.89)*	1.17 (.87-1.57)

^+^Adjusted for age, sex, race/ethnicity, marital status, diagnoses/conditions, level of ADL impairment, DNR order present, any urinary catheter use.

^1^Severity of Cognitive Impairment was measured by the Cognitive Performance Scale (CPS) – No/Mild (0, 1); Mild/Moderate (2, 3); Moderate/Severe (4, 5); Very Severe (6).

*p<.01, **p<.001, ***p<.0001.

Table 3 shows the results of our over dispersed Poisson regression that modeled the rates of hospitalizations and ACS hospitalizations. The hospitalization rate for NH residents with FTs in 2006 was 16% and the ACS hospitalization rate was 7%. Forty-four percent (242/553) of all hospitalizations in this study sample were potentially preventable and due to an ACS condition. In contrast to our ED results, severity of CI did not have a significant effect on rates of hospitalizations.

**Table 3:** Rates of hospitalizations and ACS hospitalizations according to the characteristics of nursing home residents with feeding tubes

**Characteristic**	**Hospitalization rate, no. (%)**	**Hospitalization incident rate ratios (95% confidence interval)**		**ACS Hosp rate, no. (%)**	**ACS Hosp incident rate ratios (95% confidence interval)**	
		**Unadjusted**	**Adjusted**^**+**^		**Unadjusted**	**Adjusted**^**+**^
Total	553 (15.9)			242 (6.9)		
Severity of cognitive impairment^1^						
No/Mild	138 (17.6)	1.02 (.88-1.91)	1.13 (.96-1.32)	54 (6.9)	.99 (.82-1.20)	1.18 (.97-1.44)
Mild/Moderate	167 (15.1)	Ref	Ref	85 (7.7)	Ref	Ref
Moderate/Severe	94 (16.2)	1.03 (.86-1.24)	.98 (.82-1.18)	37 (6.4)	.95 (.75-1.19)	.89 (.71-1.12)
Very severe	154 (15.4)	1.09 (.92-1.27)	.93 (.78-1.10)	66 (6.6)	.98 (.81-1.20)	.87 (.70-1.09)

^+^Adjusted for age, sex, race/ethnicity, marital status, diagnoses/conditions, level of ADL impairment, DNR order present, any urinary catheter use.

^1^Severity of Cognitive Impairment was measured by the Cognitive Performance Scale (CPS) – No/Mild (0, 1); Mild/Moderate (2, 3); Moderate/Severe (4, 5); Very Severe (6).

## Discussion

Examining a 5% random sample of Medicare beneficiaries in 2006, we found that approximately 25% of NH residents with FTs required an ED visit or hospitalization, with 44% of hospitalizations and 24% of ED visits being potentially preventable for an ACS condition. Moreover, nearly 30% of NH residents with FTs were considered “comatose” or without “discernible consciousness”, representing 20060 NH residents with FTs in the US. We further found that 42% of NH residents with FTs were dependent in all ADLs, representing 31,700 NH residents who are unable to eat, bathe, toilet, transfer, or dress.

Previous studies suggest that there are little or no benefits to FTs in cognitively impaired patients, particularly among those with advanced dementia. Evidence suggests that FTs in cognitively impaired patients do not prevent aspiration pneumonia, improve function, prevent or improve pressure ulcers, reduce the risk of infection, meaningfully improve nutritional status, decrease weight loss, improve wound healing, improve patient comfort, or decrease mortality – all the reasons commonly cited to support FT placement [6,7,10,11,20,41-47]. Among our national sample of tube-fed NH residents, we found that nearly half were classified as having at least severe or very severe CI (CPS 4–6, MMSE < 7).

Our results further indicate that the risks of hospitalization and ED visits vary by degree of CI. Tube-fed NH residents with severe CI (CPS 4–5, MMSE 5–7) are more likely to need ED evaluation than NH residents with moderate CI (CPS 2–3, MMSE 15–19) or very severe CI (CPS 6, MMSE 0–4). One possible explanation could be that NH staff, providers and families may recognize that ED visits may be especially burdensome for NH residents with profound CI and thus attempt to minimize ED visits for these residents. Alternatively, since NH residents with very severe CI are described as “comatose” or showing “no discernible consciousness”, these patients may be physically unable to become agitated. In contrast, patients with severe CI may become agitated during intercurrent illness, prompting transfers to the ED. Evidence suggests that a frequent precipitant for acute care transfers is dementia-related behavioral issues [48], which are commonly exacerbated with acute medical illness.

In contrast to our ED visit results, we found that hospitalizations did not vary significantly by degree of CI. Our results may reflect the fact that the decision to hospitalize is often based on more data such as lab tests and radiology results which often do not account for CI. In contrast, the initial decision to transfer a NH resident for an ED evaluation is based more on overall clinical impression that may implicitly incorporate a patient’s cognitive status. Alternatively, the presence of CI may actually cloud the ability of nursing staff to appropriately recognize the nature and severity of a change in health status. As a result, nursing staff may err on the side of transferring residents to the hospital for conditions that could be potentially managed in the NH.

Our ACS results suggest that severity of CI has a significant effect on rates of potentially preventable, or ACS, ED visits, but little effect on ACS hospitalizations. NH residents with no/mild CI (CPS 0–1, MMSE = 22-25) and severe CI (CPS 4–5, MMSE = 5-7) have significantly higher rates of ACS ED visits than those with moderate CI (CPS 2–3, MMSE = 15-19) or very severe CI (CPS 6, MMSE = 0-4), the latter of which is the population typically studied in this body of literature. While families and caregivers may prefer more aggressive care for acute medical problems in the earlier stages of CI, our findings suggest that the very presence of even mild CI in a NH resident with a FT may increase the risk of a potentially preventable ED visit. Prior research suggests that family members of NH residents with dementia are often unsure about the length and expected course of the illness and such uncertainty makes it difficult to determine whether an acute illness is part of a downward trajectory or a temporary, reversible setback [49].

For many cognitively impaired NH residents, a transfer to the hospital or ED likely represents a significant burden. This frail, vulnerable population is often easily confused, frustrated and frightened; tends to exhibit behavioral and psychiatric disturbances when under stress; and is often unable to communicate their needs or understand care instructions. Given the fast-paced nature of the ED and focus on rapid triage and treatment, many cognitively impaired patients may not understand why they are moved from their familiar surroundings to a strange setting with unfamiliar caregivers who must perform uncomfortable procedures such as venipuncture or FT reinsertion. Moreover, hospital transfers in this population frequently results in greater cognitive and functional decline, delirium, iatrogenic complications and death [16,17]. Thus, our finding that one in four tube-fed NH residents require hospitalization or ED visits likely represents a substantial burden for this vulnerable cognitively impaired population.

There is growing recognition of the unique and important role ED care providers can play in supporting early palliative care interventions along a patient’s disease trajectory, promoting quality of life, as well as reducing treatment costs [50-54]. Recent studies indicate that palliative interventions in the ED may improve timely provision of care, improve care outcomes, increase direct referrals to hospice, decreased lengths of stay, improve patient and family satisfaction, and reduce intensive care utilization, as well as health care costs [55-57]. Additional research is needed to evaluate the effect of such interventions with frail NH residents, such as those with feeding tubes and CI, who may frequently visit the ED.

Our results should be interpreted in light of our study’s limitations. First, we relied on administrative data and therefore lacked detailed clinical information regarding causes of ED and hospital visits. This also limited our ability to account for individual clinical characteristics. However, we were able to rely on validated scales and frequently used MDS resident-level variables to risk adjust our outcomes. Second, we were unable to determine the indication for FT placement. It is possible that some residents with moderate/severe or very severe CI received a FT for nutritional support after a stroke, trauma, or head or neck cancer for which there is some data to suggest FTs may lead to better outcomes [14,41,58]. Third, by focusing only on residents with FTs we may have selected for a population with an inherent preference towards more aggressive care, including transfers to the acute care hospital. Despite these limitations, this is the first study to describe rates of hospitalization and ED visits, including for potentially preventable conditions, among a national random sample of NH residents with FTs and different levels of CI.

## Conclusion

This study revealed that ED visits and hospitalizations are common in this vulnerable population and a significant proportion of both ED visits and hospitalizations are potentially preventable. These potentially unnecessary care transitions may lead to a premature cascade of excess disability, with higher rates of morbidity and mortality, decreased quality of life and higher health care costs. Such risks should be considered in the decision making process regarding the placement of FTs in persons with CI and palliative care interventions should be evaluated for those with frequent ED visits.

## Abbreviations

ACS: Ambulatory care sensitive; ADL: Activities of daily living; AHRQ: Agency for healthcare research and quality; CHESS: Changes in health end-stage disease and symptoms and signs; CI: Cognitive impairment; CMS: Centers for medicare and medicaid services; CPS: Cognitive performance scale; ED: Emergency department; FT: Feeding tube; IRR: Incident rate ratio; MDS: Minimum data set; MEDPAR: Medicare provider analysis and review; MMSE: Mini mental status exam; NH: Nursing home; PQI: Prevention quality indicator; SAF: Standard analytic file.

## Competing interests

The authors declare that they have no competing interests.

## Authors’ contributions

CS conceptualized and designed the study; acquired, analyzed and interpreted the data; and drafted the manuscript. NS and PG participated in the study design and coordination; helped interpret the data; and draft the manuscript. SL helped conceptualize and design the study; analyze and interpret data; and draft the manuscript. All authors read and approved the final manuscript.

## Pre-publication history

The pre-publication history for this paper can be accessed here:

http://www.biomedcentral.com/1471-2318/14/35/prepub
